# Childhood trauma and mental disorders: Exploring the relationship between trauma, immunity and psychosis

**DOI:** 10.1192/j.eurpsy.2021.1259

**Published:** 2021-08-13

**Authors:** I. Figueiredo, F. Viegas, F. Ferreira, C. Manuel

**Affiliations:** 1 Mental Health Department, Hospital Professor Doutor Fernando Fonseca, Lisboa (Amadora), Portugal; 2 Mental Health Department, Hospital Professor Doutor Fernando Fonseca, Lisboa (Amadora), Portugal; 3 Psychiatry, Hospital Professor Doutor Fernando Fonseca, Lisboa (Amadora), Portugal

**Keywords:** childhood trauma, ımmune system, psychosis

## Abstract

**Introduction:**

A relationship between childhood trauma, psychotic experiences, and psychosis is well established, although causality is not yet ascertained. There are several hypotheses linking trauma and psychosis, regarding genetic vulnerability and/or other environmental factors, possibly also mediated by psychological mechanisms. Long-term modifications to the transcriptome are likely mediated by epigenetic mechanisms. There is also growing evidence supporting an association between childhood trauma and adulthood dysregulation of the immune system, which could help clarify the relationship between trauma and mental disorders, namely psychosis.

**Objectives:**

Review evidence regarding the relationship of childhood trauma, immune system and psychosis.

**Methods:**

Literature review using Medline database.

**Results:**

The prevalence and severity of childhood trauma is characterized by both biological alterations and increased risk of experiencing symptoms of psychosis. Childhood trauma, namely through its effects on IL6 levels, may be a risk factor for schizophrenia in general. Some studies point to a direct relationship between childhood trauma, immunity and psychosis when examined along a continuum from non-clinical controls to psychotic disorders such as schizophrenia.

**Conclusions:**

For better understanding this association, these findings must be replicated in larger cohorts. If the impact of childhood trauma on immune function in adulthood does indeed contribute to psychopathology, an improved understanding of this relationship may lead to new and possibly more specific treatment options. Other clinical implications of these findings include increased emphasis in establishing more comprehensive screening of early trauma in patients with psychotic symptoms, as well as the importance of screen and follow children who report traumatic events for emergence of psychotic symptoms.

